# In response to Letter to the Editor regarding: prognostic value of age and Wassel classification in the reconstruction of thumb duplication. J Child Orthop (2013)7:551–557

**DOI:** 10.1007/s11832-014-0592-1

**Published:** 2014-05-14

**Authors:** Marisa Cabrera González, Laura M. Pérez López, Gino Martínez Soto, Diego Gutiérrez de la Iglesia

**Affiliations:** 1 Hand and Malformation Surgery Division, Department of Pediatric Orthopaedic Surgery, Sant Joan de Déu Children’s Hospital, University of Barcelona, Passeig Sant Joan de Déu, 2 Esplugues de Llobregat, ES08950, Barcelona, Spain; 2 School of Medicine, Pontificia Universidad Católica de Chile, Santiago de Chile, Chile

We are pleased to see the great interest that our study on thumb duplication has generated among the community of pediatric orthopedic surgeons. We read this Letter to the Editor with great enthusiasm as it reflects some of the interesting aspects that are still under discussion in our field. We are happy to advance the discussion on thumb duplication as it may enhance our knowledge and provide better medical management of our patients.

Let us address the writers’ concerns stated in their letter. Regarding the mean duration of follow-up in relation to the mean age at surgery, we have checked the mean follow-up (in months) for each age group at the time of their surgery. Excluding the youngest group, the follow-up durations were quite equally distributed among groups, although the oldest group had the shortest follow-up (and the most complications). This finding means that the larger number of complications in the older groups was not owed to longer follow-ups in these groups and thus not owed to the older final ages at the end of the follow-up. Even assuming that the youngest group had their final follow-up at a younger age than the rest of the groups, which also may be the reason there were fewer complications in this group, it would not contradict the fact that there was a progressive increase in the number of complications in Wassel groups II–IV (with the difference being statistically significant, *p* = 0.0002).

Also, the different numbers of complications that occur in patients of older or younger ages at follow-up did not depend on differences at 5 months of follow-up but over longer periods of time in accordance with growth spurts, damage to or asymmetrical joints and bone, or tendon imbalance, as the authors of the Letter to the Editor mention.

Although our mean follow-up was similar to those in other publications, we agree with the Letter’s authors that this issue should be addressed and solutions discussed. As patients lost to follow-up may significantly decrease the groups’ mean age, perhaps it would be better to include only patients up to preadolescence in these studies.

Regarding the Tada scoring system, we did not make any reference to there being only one system to describe outcomes of radial polydactyly. We agree that choosing one classification system or another may influence the final results and conclusions. For this reason, and considering that Tada has been the validated functional scale used in most of the internationally published papers on thumb duplication, we believed that it made sense to use the same system to render our results comparable to those of previous studies. By using the same scale (Tada), results can be interpreted using the same criteria as were used in various other studies on thumb duplication.

There was also a point made by the authors of the Letter regarding outcomes. They noted that the patient’s perception of the hand’s appearance is not addressed by the Tada system. We did note in the article (in regard to the importance of performing the surgery early) that the patient needs “to develop a normal body image … to facilitate the social integration of the patient when they start school”. Thus, we agree that an aesthetic consideration should be added to whatever different system we use in the future.

In that sense, after the publication of our paper, we read a relevant article about the classification systems for polydactyly [1]. Dijkman et al., under the supervision of Professor S. Hovius, concluded that the Japanese Society for Surgery of the Hand (JSSH) has provided the most reliable outcome scores for radial polydactyly in regard to scientific evaluation of this pathology. Based on their article, we predict that future studies on thumb duplication will use the JCS, abandoning (for the most part) the previous tendency to use Tada. We congratulate Hovius’ team for their excellent work and for the relevant conclusions of their study. Future studies on thumb duplication will be able to provide more extensive and credible information in the future because of their article.

Regarding our choice of classification, Wassel described his classification system in 1969 using Flatt’s series of patients [2]. From that time to the present day, triphalangism has been progressively considered a situation different from that of thumb duplication and requires a different approach. Its treatment is widely described in Green’s Operative Hand Surgery, 6th Edition. For this reason, we used the Wassel classification as modified by Egawa [3, 4], which describes type VII as a floating thumb.

When describing our results, we did not present definitive conclusions. As noted in the text, we believed that in future efforts thumb duplication should undergo more rigorous statistical and methodological study. We are aware of the limitations of our study (which are addressed in the article) and thus encourage other authors to develop more rigorous studies. Our conclusions were not presented as either definitive or unquestionable but as initial statistical analyses that may show a new way to obtain more reliable data on thumb duplication. The study was detailed, and the statistical analysis was clear. This study was the first in the literature to provide statistical data on thumb duplication. With it, we tried to suggest an approach to future studies with stricter conditions. The next analysis might be prospective with longer follow-ups, thereby being better both methodologically and statistically—providing more useful results. Taking into account that the prognostic value of age and the Wassel classification for reconstruction to correct thumb duplication methodologically have led others to write papers on the subject that are simply descriptive evaluations, we think that our published statistical study is a good contribution to the analysis of treatment for thumb duplication.

We again thank the authors of this Letter to the Editor for their interest in our study and detailed evaluation of our results. The issues that are presented are interesting and should encourage the community of pediatric hand surgeons to continue study into this interesting pathology. There remains much work to be done.
